# On the Dissociation of Word/Nonword Repetition Effects in Lexical Decision: An Evidence Accumulation Account

**DOI:** 10.3389/fpsyg.2016.00215

**Published:** 2016-02-19

**Authors:** Manuel Perea, Ana Marcet, Marta Vergara-Martínez, Pablo Gomez

**Affiliations:** ^1^Estructura de Recerca Interdisciplinar de Lectura (ERI-Lectura), Universitat de ValènciaValència, Spain; ^2^Basque Center on Brain, Cognition, and LanguageDonostia, Spain; ^3^Department of Psychology, DePaul UniversityChicago, IL, USA

**Keywords:** lexical decision, diffusion model, repetition, word processing, RT distributions

## Abstract

A number of models of visual-word recognition assume that the repetition of an item in a lexical decision experiment increases that item's familiarity/wordness. This would produce not only a facilitative repetition effect for words, but also an inhibitory effect for nonwords (i.e., more familiarity/wordness makes the negative decision slower). We conducted a two-block lexical decision experiment to examine word/nonword repetition effects in the framework of a leading “familiarity/wordness” model of the lexical decision task, namely, the diffusion model (Ratcliff et al., 2004). Results showed that while repeated words were responded to faster than the unrepeated words, repeated nonwords were responded to more slowly than the nonrepeated nonwords. Fits from the diffusion model revealed that the repetition effect for words/nonwords was mainly due to differences in the familiarity/wordness (drift rate) parameter. This word/nonword dissociation favors those accounts that posit that the previous presentation of an item increases its degree of familiarity/wordness.

## Introduction

The study of how words are learned, processed, and identified is a central topic in cognitive psychology and cognitive neuroscience (see Carreiras et al., 2014, for a recent review). To examine the processes underlying word recognition, researchers employ a number of different techniques, of which the most widespread is the lexical decision task (LDT). In a typical LDT experiment, a letter string is presented on the center of a computer screen and participants have to decide, as rapidly and accurately as possible, whether the letter string is a word or not by pressing a key. In behavioral experiments, the dependent variables are the response time (RT) and the accuracy.

A shared assumption in many influential models of the LDT is that lexical decision responses are, at least in part, driven by the familiarity/wordness of the stimulus. The first model to explicitly implement this assumption is Chumbley and Balota (1984) two-process model. Balota and Chumbley posited that, in the early stages of processing, “the subject makes a quick check to determine if the stimulus is producing any meaning or is very familiar, that is, ‘Have I seen this stimulus frequently?”’ (p. 352). If the familiarity/wordness of the letter string is higher than a given upper criterion (i.e., the word TIME), there will be a fast “word” response, whereas if the letter string is lower in familiarity/wordness than a lower criterion (i.e., the illegal nonword XZTA), there will be a fast “nonword” response. The stimuli whose familiarity falls between the two criteria will require further, more analytic processing. With different flavors, the idea of familiarity/wordness driving the decision process in the LDT has been employed in a number of influential models of visual-word recognition (e.g., multiple read-out model [MROM], Grainger and Jacobs, 1996; dual-route cascaded [DRC] model, Coltheart et al., 2001; REM-LD model; Wagenmakers et al., 2004a; diffusion model; Ratcliff et al., 2004; Bayesian reader model, Norris, 2006).

A fairly straightforward prediction of “familiarity/wordness” models of the LDT is the dissociation of word and nonword repetition effects. As the initial presentation of a stimulus would boost its familiarity/wordness, repetition would benefit lexical decision to words, whereas it would hinder lexical decision to nonwords. There is some empirical evidence supporting this dissociation. Balota and Spieler (1999) reported a facilitative repetition effect for words and an inhibitory repetition effect for nonwords in a two-block experiment. In the first block, participants performed a rhyme judgment task. For instance, the response to pairs of stimuli such as “where-air” or “tube-doob” would be “yes,” whereas the response to pairs of stimuli such as “gritty-carry” or “strong-hing” would be “no.” In the second block, participants performed a lexical decision task. Half of the items were repeated from the previous block (e.g., “air,” “doob,” “carry” or “hing”) whereas the other half of items was not repeated. That is, they compared the response times of the repeated vs. the nonrepeated stimuli (i.e., practice and repetition were not confounded). Balota and Spieler reported that, for words, lexical decision responses were faster for repeated than for nonrepeated items (the magnitude of the effect was 17 ms for high-frequency words and 30 ms for low-frequency words), whereas for nonwords, lexical decision responses were slower for repeated than for nonrepeated items (the magnitude of the effect was −11 ms). However, there was a potential shortcoming in the Balota and Spieler (1999) experiment: the rhyming task in the initial block could have induced an unwanted consequence. Each nonword was always associated to a word (e.g., “doob” would be presented just after “tube”), such that the memory trace for “doob” may have been associated to the word “tube,” increasing its familiarity/wordness (see Wagenmakers et al., 2004a, for a similar reasoning). Because of this association, the representation of the nonword would be even more wordlike in the second presentation as an artifact of the procedure, thus enhancing the inhibitory repetition effect for nonwords. A more direct test of the dissociative repetition effects of words vs. nonwords in the LDT would require blocks in which there are no explicit associations between nonwords and words. The present experiment aims to fill this gap. Furthermore, we provided fits from a mathematical “familiarity/wordness” model of the LDT, the diffusion model (Ratcliff et al., 2004). We focused on the diffusion model because other computational/mathematical models have not focused on the effects on repetition effects of words vs. nonwords in the LDT (e.g., MROM, DRC model, Bayesian reader model) or have exclusively focused on accuracy (REM-LD model). But before describing the experiment, we offer a brief account of the diffusion model, and how it can account for word and nonword repetition effects in the LDT.

In the diffusion model account of the LDT, there is an accumulation of noisy evidence over time toward one of two response boundaries (e.g., “word” and “nonword”; Ratcliff et al., 2004; see also Ratcliff et al., 2004, 2012; Gomez et al., 2007, 2013; Wagenmakers et al., 2008; Gomez, 2012; Perea and Gomez, 2012; Gomez and Perea, 2014). A fundamental parameter in the model is the drift rate (i.e., the rate of accumulation of evidence). In the LDT, the drift rate is a direct function of how familiar/wordlike the letter string is. The more familiar/wordlike a letter string is, the faster the evidence reaches the “word” boundary, whereas the less familiar/wordlike a letter string is, the faster the evidence reaches the “nonword” boundary. Ratcliff et al. (2004) indicated that the decision process makes use of several sources of information (e.g., orthographic, phonological, semantic) that are combined into a single quantity (i.e., the drift rate parameter) that reflects the degree of “wordness” (i.e., familiarity/wordness) of the letter string. In a diffusion model account of the LDT, the effects of word-frequency and repetition are “simply to alter the amount and kind of information contributing to the degree of wordness that drives the decision process and nothing more” (Ratcliff et al., 2004; p. 76). For instance, a high-frequency word (e.g., HOUSE) produces a larger degree of familiarity/wordness than a low-frequency word (e.g., DIURNAL). In turn, a consonant string (e.g., FGMTN) produces a lower degree of familiarity/wordness than an orthographically legal nonword (e.g., WOUSE). Similarly, a repeated word would produce a larger degree of familiarity/wordness than a new word. Importantly, when two experimental conditions differ in drift rate, the diffusion model not only predicts that the condition with a smaller drift rate produces longer (on average) lexical decision times than the condition with a greater drift rate, but also that the difference between conditions increases in the higher quantiles of the RT distributions (see Figure 2 in Ratcliff et al., 2004, for illustration).

Lexical decision data generally support the basic tenets of the diffusion model. Ratcliff et al. (2004; see also Gomez et al., 2007; Gomez and Perea, 2014, for similar evidence; but see Norris, 2009, for criticism) found that the word-frequency effect (i.e., the difference between the RTs of low- vs. high-frequency words) in lexical decision was substantially greater at the tail of the RT distributions (e.g., 0.7 and 0.9 quantiles) than at the leading edge of the RT distributions (0.1 quantile). Likewise, Ratcliff et al. (2004, Experiment 8) found that the repetition effect for words, defined as the difference between the initial and second presentation of the same word, was substantially larger at the tail of the RT distributions (e.g., 0.7 and 0.9 quantiles) than at the leading edge of the RT distributions (0.1 quantile). Fits from the diffusion model corroborated that the differences across conditions (high- vs. low-frequency words; words' first vs. second repetition) occurred mainly in the drift rate parameter.

While the data from the word stimuli in Ratcliff et al.'s (2004) Experiment 8 are entirely consistent with a diffusion model account, the story is more complex than originally thought. Ratcliff et al. (2004) found a non-significant small inhibitory repetition effect for nonwords in the latency data (683 vs. 686 ms in the first and second presentations, respectively; *p* = 0.08) that was accompanied by a facilitative repetition priming effect in the accuracy data (6.0 vs. 7.9 % of errors in the first and second presentations, respectively; *p* < 0.01). As stated earlier, if the repetition of any stimulus produces an increase in the degree of familiarity/wordness, one would have expected not only a facilitative repetition effect for words, but also an inhibitory repetition effect for nonwords. Furthermore, this inhibitory effect should be manifest in the higher quantiles of the RT distribution, as it would reflect a change in drift rate. However, the RT data at the 0.9 quantile in the Ratcliff et al. (2004) experiment only showed a −2 ms inhibitory nonword repetition effect. What we should note here is that the design employed by Ratcliff et al. (2004) was not optimal to detect repetition effects, as there was a confound with a general effect of practice (see Dutilh et al., 2009, for discussion of practice effects in lexical decision). In the Ratcliff et al. (2004) Experiment 8, the repetition effect was defined as the difference between the first and second presentation of the same item. Therefore, the amount of practice in the task was always higher by the time of the second presentation of the nonword than by the time of the initial presentation. In other words, the nonword repetition effect in Ratcliff et al.'s (2004) Experiment 8 could have been confounded by the fact that the second presentation occurred later (i.e., when the participant had more practice in the task) than the first presentation, and the two effects could have canceled out each other (i.e., a facilitative practice effect vs. an inhibitory nonword repetition effect). As indicated earlier, Balota and Spieler (1999) found a facilitative repetition effect for words and an inhibitory repetition effect for nonwords using a classic two-block experimental design that avoided that confound. Importantly, for both words and nonwords, the magnitude of the repetition effect increased across quantiles (see Figure 4 in Balota and Spieler, 1999), as the diffusion model would have predicted. Likewise, Wagenmakers et al. (2004b) found an inhibitory repetition priming for nonwords in the error rates in a signal-to-respond LDT with relatively short variable time intervals (between 350 and 600 ms). Importantly, Zeelenberg et al. (2004) found an inhibitory nonword repetition effect in the error rates when speed was stressed in the instructions (i.e., all response times should be below 500 ms), but not when accuracy was stressed. While the data reported by Wagenmakers et al. (2004b) and Zeelenberg et al. (2004) are consistent with the idea that a repeated nonword is more familiar/wordlike than a non-repeated nonword, these experiments focused exclusively on the error rates. In the current experiment, we opted for using standard lexical decision instructions, as this allowed us to examine the RTs as the main dependent variable.

It is important to note here that Zeelenberg et al. (2004) argued that stressing speed maximized the chances to make lexical decisions on the basis of familiarity/wordness rather than from a more elaborate (post-access) analysis of the stimuli. Indeed, there is some evidence that, under some circumstances, nonword repetition effects can be facilitative in the LDT (e.g., Logan, 1990). This facilitative nonword repetition effect has often been associated to the retrieval of specific instances (e.g., “JUGPE is a nonword”) that would help the lexical decision process. What is the origin of this apparent discrepancy? As Wagenmakers et al. (2004b) discussed, the nonword repetition effect in the LDT can originate from two different and opposing processes. On the one hand, there may be a quick, fast-acting process in which familiarity/wordness drives lexical decisions. This would facilitate the responses to repeated words, but have a detrimental effect on the processing of repeated nonwords—this view would be consistent with “familiarity/wordness” models of the LDT. On the other hand, there may be a slow-acting, episodic retrieval of information that would presumably benefit *both* words and nonwords—this would be consistent with Logan's (1990) instance theory. As Logan (1990) argued, “both words and nonwords generate associations” and “repetition would strengthen associations for both words and nonwords.” (pp. 26–27).

In the present lexical decision experiment, we employed two blocks. In the first block, participants were presented with 200 trials (100 word trials and 100 nonword trials); in the second block, which occurred immediately after finishing the initial block, participants were presented with 200 trials (100 word trials and 100 nonword trials). Half of the stimuli in the second block had already been presented in the initial block (i.e., 50 repeated words and 50 repeated nonwords) whereas the other half was composed of new stimuli (i.e., 50 nonrepeated words and 50 nonrepeated nonwords)—two lists were randomly created to counterbalance the stimuli. We maximized the chances to capture fast-acting (familiarity-driven) decision processes rather than post-access checking/verification processes that may occur in the LDT when stressing accuracy or when using irregular/unfamiliar words. To that end, all the word stimuli were of very high frequency (*M* = 195 per million words).

In sum, the goal of the present lexical decision experiment was to examine the predictions of a “familiarity/wordness” model of the LDT (i.e., the diffusion model) concerning the dissociative effects of repetition for words and nonwords. To that end, we employed a classic experimental two-block design with standard instructions (i.e., respond as fast as possible while trying to keep low the error rate). In the framework of those models that assume that “familiarity/wordness” is accumulated over time and drives the decision process, the predictions are clear: If the first presentation of a stimulus (word/nonword) produces an increase in the quantity of familiarity/wordness, then one would expect faster RTs for repeated words than for nonrepeated words. Conversely, RTs should be slower for repeated nonwords than for nonrepeated nonwords. Furthermore, these repetition effects should occur to a greater degree in the higher quantiles of the RT distribution (i.e., 0.7 and 0.9 quantiles) than in the leading edge of the RT distribution (0.1 quantile). We can predict, therefore, that a diffusion model implementation in which the drift rate parameter is free to become more positive as a function of repetition should account for the difference between the repeated and non-repeated items. Alternatively, if lexical decision responses to words/nonwords are typically due to the retrieval of previous episodes, as Logan's (1990) instance theory would predict, one would expect a facilitative repetition effect not only for words, but also for nonwords.

## Materials and methods

### Participants

A total of twenty-four students from the University of Valencia took part in the experiment voluntarily. All of them had normal or corrected-to-normal vision and were native speakers of Spanish. The Experimental Research Ethics Committee of the Universitat de València (Spain) approved this experiment. All participants gave written informed consent in accordance with the Declaration of Helsinki.

### Materials

One hundred Spanish words, all of them nouns, were selected from the EsPal database (Duchon et al., 2013). All these words were of high frequency (*M* = 195 per million words in the subtitle corpus of EsPal; range 104–452). The mean number of letters was 6.1 (range: 5–8), the mean number of orthographic (one-letter different) neighbors was 2.1 (range: 0–14), the mean Levenshtein distance (OLD20) was 1.7 (range: 1.0–2.9). We also created 100 nonwords with Wuggy (Keuleers and Brysbaert, 2010), so that the number of letters, number of syllables, subsyllabic structure, and transitional probabilities were matched with those of the word stimuli. The list of words and nonwords is available in the Appendix. For the sake of the main manipulation (item repetition), 50 filler (high-frequency: were above 83 occurrences per million) words and 50 filler nonwords were selected, similar in length and in the other psycholinguistic variables to the experimental stimuli. Two sets of materials (Set 1 and Set 2) were constructed and presented across Block 1 and Block 2. For both sets, Block 2 consisted of the same 200 experimental items (100 words and 100 nonwords). Half of those items (50 words and 50 nonwords) were randomly chosen for inclusion in Block 1 for Set 1 (together with 50 filler words and 50 filler nonwords), and the other half were chosen for Set 2 (together with 50 filler words and 50 filler nonwords). Thus, each block contained 200 items (100 words and 100 nonwords).

### Procedure

Participants were tested individually in a quiet room. Presentation of the stimuli and recording of reaction times were controlled by the DMDX software (Forster and Forster, 2003). On each trial, a fixation point (“+”) was presented for 500 ms on the center of the screen. Then, a lowercase letter string was presented until the participant responded or 2 s had elapsed. Participants were instructed to press the “sí” (“yes”) key if the letter string was a legitimate Spanish word and to press “no” if the letter string was not a word. Subjects were instructed to make this decision as quickly and as accurately as possible. After the participant had finished Block 1, the computer program indicated that the same instructions applied to Block 2. Each subject received a total of 12 practice trials prior to Block 1, and 4 practice trials prior to Block 2. Stimulus presentation in each block was randomized for each participant. The whole session lasted approximately 12–14 min.

## Results

### Empirical findings

Given our research goals, only the responses in block 2 were analyzed. Incorrect responses (4.0%) and reaction times shorter than 250 ms or longer than 1500 ms (less than 0.4%) were excluded from the RT analyses. The mean RT and the percentage of errors on the words and nonwords in each experimental condition are displayed in Table 1.

**Table 1:** **Mean lexical decision times (RT, in ms) and percent error (ER) on words and nonwords in Phase 2 of the Experiment**.

	**Repeated**		**Nonrepeated**		**Nonrepeated—Repeated**	
	**RT**	**ER**	**RT**	**ER**	**RT**	**ER**
Words	513	2.9	531	3.7	18 [8.7, 28.3]	0.8 [−0.7, 2.3]
Nonwords	597	4.7	583	4.5	−14 [−24.8, −3.3]	−0.2 [−1.8, 1.1]

*The 95% confidence intervals for the repetition effect are presented between brackets*.

**Figure 1:**
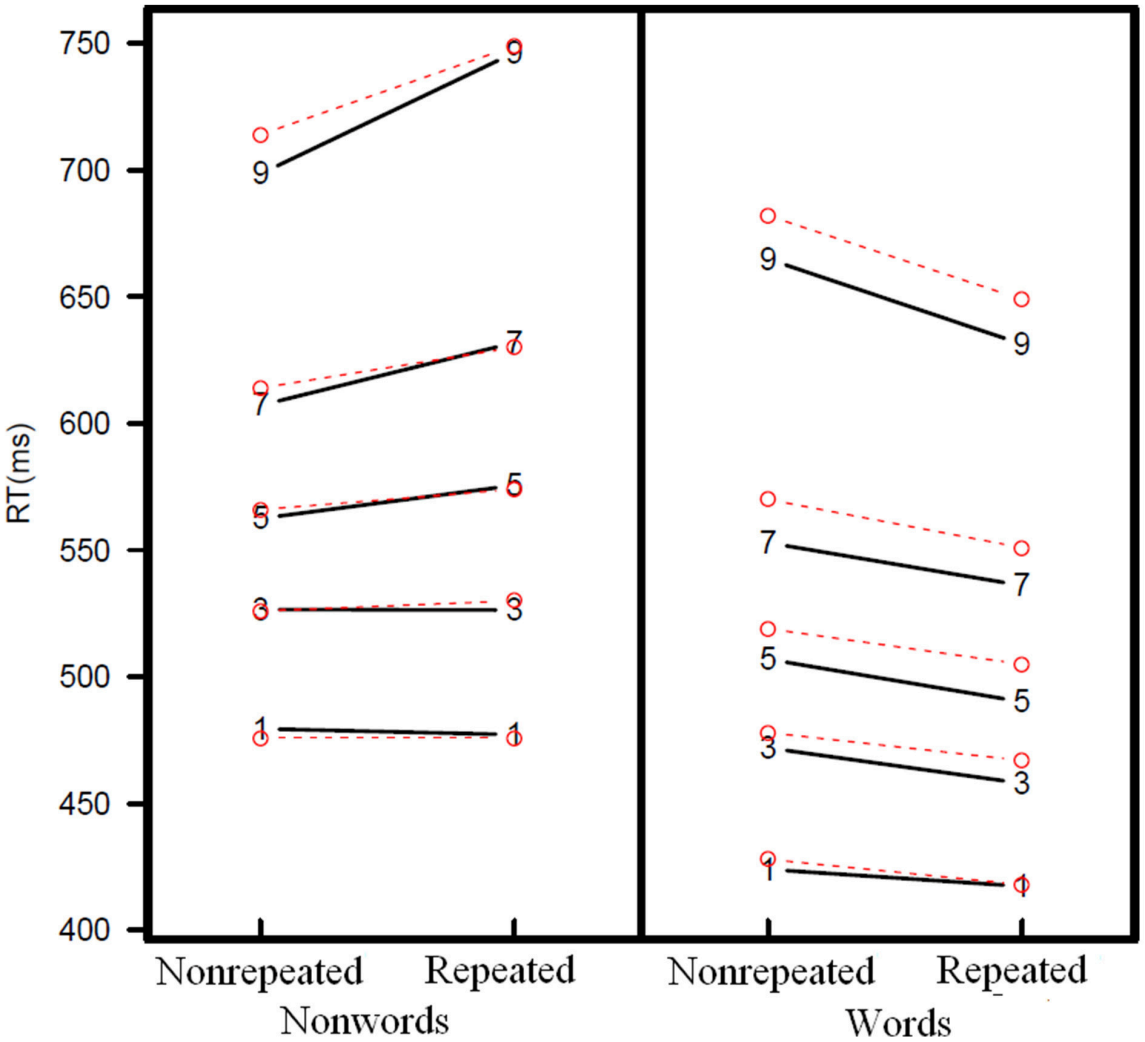
**Latencies at five quantiles for the grouped data (vincentiles)**. The points represent (from bottom to top) the 0.1, 0.3, 0.5, 0.7, and 0.9 quantiles. The digits represent the empirical data, and the points represent the model fits.

To examine the effects of the two fixed factors (i.e., repetition [repeated, nonrepeated] and lexical status [word, nonword] on the RTs, we conducted linear mixed-effects models using R (lme4 package; Bates et al., 2014). To account for the positive asymmetry of the RT data (see Figure 1), we used −1000/RT as the dependent variable in the latency analyses—note that this transformation maintains the direction of the observed effects. There were 4585 data observations in the RT data. The *p* values were obtained from the lmerTest package (Kuznetsova et al., 2014). The model also included random intercepts for subjects and items, as well as the by-subject random slopes for repetition^*^lexical status—the model with the maximal random structure model did not converge. The statistical analyses for the error data were modeled using the *glmer* function in the *lme4* package, as errors are binary values (1 = correct, 0 = incorrect).

The statistical analyses on the correct RTs showed that participants responded to more rapidly to words than to nonwords, *t* = 9.17, *p* < 0.001. The main effect of repetition was not significant, *t* = 1.4, *p* > 0.15. More important, the interaction between lexicality and repetition was significant, *t* = 3.71, *p* = 0.001. (Unsurprisingly, the classical *F*1 and *F*2 analyses yielded the same interaction as that reported here.) This interaction reflected a facilitative repetition effect for words, *t* = 3.98, *p* < 0.001, and an inhibitory repetition effect for nonwords, *t* = 2.53, *p* = 0.018.

The statistical analyses on the error rates did not show any significant effects.

### Modeling

A simplified version of the diffusion model was fit to the data from the present experiment. A more comprehensive description of diffusion modeling of lexical decision data can be found elsewhere (e.g., Ratcliff et al., 2004; Gomez, 2012; Gomez et al., 2013). As indicated in the Introduction, when applied to the LDT, the model assumes that there is noisy accumulation of evidence triggered by the stimulus. This evidence is accumulated until a “word” or a “nonword” decision is reached. Importantly, the model parameters can be linked to underlying psychological processes (see Gomez, 2012): (i) The drift rate parameter (υ) relates to the rate of information extraction, akin to the lexical activation of an item; (ii) the boundary separation parameter (*a*) relates to response caution, or how much evidence is needed to make a response; (iii) the starting point of the diffusion process (*z*) relates to the *a priori* response biases in terms of how one response might require less evidence than the other; and (iv) the time of encoding and response parameter (*T*_*er*_) relates to the time taken by the encoding of the sensory information and the motor execution of the response.

### Modeling strategy

The main parameter of interest within the present article is the drift rate, as it maps into the familiarity/wordness concept that we have described above. In addition, we explored the behavior of the time of encoding/response parameter (*T*_*er*_), as previous research suggests that priming (Gomez et al., 2013) and lexicality (Gomez and Perea, 2014) affect this parameter of the model: primed words are faster to encode, and so are words relative to nonwords.

Previous applications of the diffusion model demonstrated that fitting the model to each participant's data provides similar conclusions as fitting the model to grouped data (also known as vincentiles; Vincent, 1912; see Gomez et al., 2013, for discussion); in the present article, we fitted the model to the grouped data. A limitation of the present experiment is that we only had one category of words (high-frequency words), and one category of nonwords, and in both cases the accuracy was quite large. This is problematic for diffusion modeling because parameters are best constrained by the error and correct responses across several conditions that need to be accounted for simultaneously (e.g., Gomez and Perea, 2014, employed up to nine types of stimuli). Given this limitation, we decided to impose *a priori* constraints on the parameters of the model while carrying out the fits. We assumed that a principled way to do this would be to constrain the model by setting the values of six of the nine diffusion model parameters to values obtained in previous experiments conducted with the same population of participants (i.e., undergraduates from the Universitat de València). Namely, we employed the values obtained by Gomez and Perea (2014) for the variability parameters of the model (see Table 2), and for the starting point of the diffusion process (as defined by location of the starting point relative to the boundary separation). In the present implementation, we allowed the boundary separation to change from the Gomez and Perea standard, but it was kept constant across all conditions as is common in diffusion model applications. Note that we still assumed that there is variability in starting point (parameter sz); in other words, participants could adjust the starting point due to the sequence of trials or due to post-error strategies (see Kinoshita et al., 2011 for a demonstration of trial to trial changes in decisional criteria).

**Table 2:** **Diffusion model parameters**.

**Stimuli**	**Repeated**	**υ**	***a***	***z***	**T_er_**	**η**	**S_z_**	**S_t_**	**p_0_**
Words	No	0.32	0.10	0.054	0.406	0.034	0.002	0.14	0.005
Words	Yes	0.36			0.400				
Nonwords	No	−0.37			0.445				
Nonwords	Yes	−0.31			0.439				

*The values for the a, z, η, S_z_, S_t_, and p_0_ parameters of the model were kept constant for all conditions in the experiment*.

To summarize, the only free parameters across conditions were the drift rates (υ) and the encoding time (as captured by the *T*_*er*_ parameter).

### Quality of the fits

Visual inspection of Figure 1 shows that the constrained diffusion model is successful at capturing the results of this experiment. In particular, the model nicely accounts for the RT data. Although in general terms, the accuracy levels are also adequately accounted for, the model does a better job for words than for nonwords (for non-repeated words data: 0.963 vs. model: 0.968, and for repeated words data: 0.971 vs. model: 0.979). For nonwords the model predicts a reduction in accuracy for repeated items relative to unrepeated items of 0.024, which in the data is of only 0.002 (for non-repeated nonwords data: 0.955 vs. model: 0.966, and for repeated words data: 0.953 vs. model: 0.942)

### Parameter behavior

The behavior of the parameters is consistent with previous applications of the diffusion model. First, the drift rate for both words and nonwords becomes more positive as a function of repetition. Keep in mind that the drift rate maps onto the familiarity/wordness dimension. For words, this helps performance, as words become more familiar and hence the evidence toward the word boundary is accumulated at a faster rate. For nonwords, on the other hand, this hinders performance, as familiarity makes negative decisions slower. Second, the behavior of the *T*_*er*_ parameter suggests that repetition might facilitate the encoding process. This effect is rather small (6 ms for both words and nonwords), but it is worth mentioning only because it matches the behavior of this parameter in priming tasks (Gomez et al., 2013) and it provides us with a useful intuition that might explain why finding effects of priming (repetition, or masked) for nonwords can be challenging: there is a facilitation on encoding due to previous exposure, and an inhibition in the decision process. The interplay of the encoding time and the drift rate for nonwords produces an interesting pattern of results: the faster responses (the lower quantiles) show some small facilitation for repeated nonwords, but the slower responses (the higher quantiles) show an inhibition for repeated nonwords (see Figure 1). While the 6 ms of difference between the values for *T*_*er*_ for repeated and non-repeated items is rather small, it allows the model to account for a qualitative feature of the data: The fastest responses for both words and nonwords have shorter RTs than what a drift/only model implementation predicts. In fact, the implementation of the model presented here (with drift and *T*_*er*_ as free parameters), yields a 20% better fit than a model in which only drift rate is allowed to vary.^1^

## Discussion

The results of the present lexical decision experiment are straightforward. While repeated words are responded to faster than the unrepeated words, repeated nonwords are responded to more slowly than the nonrepeated nonwords. Furthermore, the magnitude of the repetition effect (nonrepeated minus repeated) increased across quantiles for both words and nonwords (see Figure 1). This pattern of repetition effects is consistent with changes in the drift rate parameter in a diffusion model (i.e., a “familiarity/wordness” model of the LDT): An increase in familiarity/wordness facilitates responses to the repeated words (i.e., a repeated word has a higher amount of familiarity/wordness, thus facilitating reaching the “word” boundary), but it hinders the responses to nonwords (i.e., the repeated nonword has a higher quantity of familiarity/wordness, thus making it more difficult to reach the “nonword” boundary). Indeed, fits from the diffusion model showed that the obtained word/nonword repetition effects could be successfully modeled as a function of drift rate (i.e., the parameter of the model that is based on familiarity/wordness).

Therefore, the present word/nonword dissociation in repetition effects favors those accounts that, as the diffusion model, posit that the previous presentation of an item increases its degree of familiarity/wordness in lexical decision (e.g., two-process model: Chumbley and Balota, 1984; Balota and Spieler, 1999; REM-LD model: Wagenmakers et al., 2004a, for similar predictions). Moreover, at an empirical level, the present data generalize the dissociative effect of word/nonwords repetition priming reported by Balota and Spieler (1999) with a classic two-block lexical decision experiment.

A remaining question is to what degree the inhibitory repetition effect for nonwords can be associated to the preactivation of similarly spelled words. Previous research is not conclusive. In a signal-to-respond procedure with the LDT, Wagenmakers et al. (2004b; Experiment 3) found an inhibitory repetition effect for nonwords which was of similar magnitude for “one-letter replaced” nonwords and “two-letter replaced” nonwords. As indicated in the Material and Methods section, all the nonwords in the current experiment were created with the same number of syllables, subsyllabic structure, and transitional probabilities as those for the words. However, there were no specific constraints as to whether the nonwords had a specific number of similarly spelled words (i.e., orthographic neighbors). Fifty-five of the one-hundred nonwords had no orthographic neighbors, and the remaining 45 nonwords had at least one orthographic neighbor—this included one-letter replacement neighbors, addition-letter neighbors, deletion-letter neighbors. We conducted a post hoc analysis to examine whether the magnitude of the inhibitory repetition effect for nonwords was greater for the “friendly” nonwords than for the “hermit” nonwords. However, the repetition effect was similar for the two types of nonwords (−16.7 vs. −13.5 ms, respectively). Therefore, as occurred with the Wagenmakers et al. (2004b) experiment, the similarity between the actual nonword with other words did not seem to drive the inhibitory repetition effect for nonwords. Nonetheless, we acknowledge that additional research is necessary to examine this issue in further detail (i.e., with a more extreme manipulation; for instance, do nonwords that resemble a high-frequency word produce a greater repetition effect than “hermit” nonwords?).

What are the implications of the present findings? The current data offer ample support for a diffusion model account of the LDT and, in general, for those accounts that assume that some form of familiarity/wordness is responsible for the dissociative repetition effects for words and nonwords (e.g., two-process model, Chumbley and Balota, 1984; REM-LD model, Wagenmakers et al., 2004a). Furthermore, the presence of an inhibitory nonword repetition effect in a lexical decision task with standard instructions poses problems for those models that assume that repetition effects are positive in nature (e.g., due to the retrieval of specific episodes; see Logan, 1990). As indicated in the Introduction, Logan (1990) claimed that repetition effects should behave similarly for words and nonwords—in fairness to Logan, it is important to stress that their experiments were aimed at examining the relationships between repetition and automaticity. What we should also note here is that, in the present experiment—as in the Balota and Spieler (1999) experiment—we employed a classic two-block experiment design in which half of the items from the first block were repeated on just one occasion in the second block, whereas in the Logan (1990) experiments there were multiple repetitions using relatively short lags. As Balota and Spieler (1999) pointed out, it is possible that participants in experiments with multiple repetitions of the same stimuli employ quite different strategies than those in two-block experiments. For instance, Balota and Spieler (1999) suggested that repetition effects in two-block designs could be ascribed to long-term priming, whereas repetition effects with multiple repetitions could be more short-lived (see Ratcliff et al., 1985). While theoretically important, a discussion of this issue would be beyond the scope of the present paper.

The present work is consistent with “familiarity/wordness” models of visual-word recognition in the LDT. To summarize, we have shown a dissociation of the repetition effect for words and nonwords (i.e., facilitative vs. inhibitory) in a two-block lexical decision experiment with standard instructions. Fits from a leading “familiarity/wordness” model (i.e., Ratcliff's diffusion model) revealed that the dissociative repetition effect for words and nonwords could be accounted for by variations of the degree of familiarity/wordness of the words and nonwords. In addition, our analysis indicates that there can be a dissociation between encoding and discriminability in the decision process: repetition facilitates the encoding of nonwords, but makes them less nonword-like.

## Author contributions

MP, AM, MV, and PG designed the experiment. AM conducted the experiment. MP and PG analyzed the data. MP, AM, MV, and PG wrote the manuscript.

### Conflict of interest statement

The authors declare that the research was conducted in the absence of any commercial or financial relationships that could be construed as a potential conflict of interest.

## Footnotes

^1^The fitting algorithm for the diffusion model was the one described by Ratcliff and Tuerlinckx (2002). This routine minimizes the difference between the expected (model prediction) and the empirical frequencies of responses that occur for each response (“word” and “nonword”) within the bins bound by RTs at the empirical quantiles using the function: (Expected–Observed)^2^/Expected. For the drift + *T*_*er*_ model this quantity was 62.8, while for the drift alone model this quantity was 73.5. See https://gist.github.com/pgomez1dpu/485e535d99ecb2d52b43 for an example. The processing of the data and the generation of the figures for the present article can be found at http://gist.github.com/pgomez1dpu/9178f432baac58324a58.

## Appendix

### Experimental stimuli

*Words*: número, orden, hermano, profesor, cuerpo, capitán, agente, línea, barco, final, atención, clase, cuarto, permiso, abogado, equipo, sueño, camino, teléfono, frente, oficina, cambio, compañía, historia, poder, palabra, mensaje, película, policía, ciudad, hospital, medio, mitad, campo, centro, control, minuto, gobierno, hambre, familia, juego, secreto, cielo, punto, fuego, placer, resto, pregunta, sistema, gusto, realidad, asesino, comida, coche, perro, maestro, vista, culpa, escuela, perdón, guerra, chico, marido, fuerza, prueba, puerta, avión, mente, forma, razón, asunto, viaje, sangre, libro, semana, traje, grupo, doctor, hotel, ayuda, miedo, sentido, negocio, música, vuelta, corazón, carta, futuro, ejército, señora, pueblo, sitio, problema, médico, llamada, dolor, cariño, cabeza, fiesta, persona.

*Nonwords*: núcena, erdin, nerlino, trovecor, cuesbo, cavatás, adiste, níleo, ranco, vilal, amarsión, flane, ciunto, pasbiso, amibada, ezuefo, ruejo, cacica, velíboco, cronte, ojenica, campia, cosmabío, nastorio, momer, malitra, ranvaje, melétuva, momecío, cipcad, hempatal, pemio, pitag, cadmo, cesfro, condrel, sivuro, fopiarno, nample, gasalia, jaubo, tellero, cauro, murto, vuemo, planor, sasto, trevonta, dosteza, hunto, peadidol, amisica, covado, mogle, pibro, paontro, lusta, cunza, estuisa, mergón, nuella, trido, sacida, fuenva, grueja, pialta, adial, muste, fonga, bavón, acisto, daije, tanfle, ficlo, tecara, chañe, brubo, diltor, horol, aucta, miaco, dintado, nedosia, rúnaca, dialta, cocigón, macta, nucura, ehársiso, tevoro, muebro, ririo, trolleba, séviso, blasida, goror, cacapo, cacepo, luesta, pasdona.

### Filler stimuli (block 1)

*Words*: silencio, programa, servicio, tienda, ataque, prisa, regalo, derecha, deseo, suelo, navidad, cámara, carrera, relación, libertad, novio, papel, basura, peligro, decisión, cárcel, misión, destino, broma, cerebro, caballo, reunión, edificio, sargento, contacto, canción, infierno, carne, locura, sorpresa, norte, iglesia, nombre, madre, lugar, amigo, mundo, dinero, padre, trabajo, gente, noche, hombre, tiempo, verdad.

*Nonwords*: prodriga, sasnicio, saunda, acapio, frina, debilo, recetra, selea, raulo, hapadad, cáviro, calleto, pelariad, vidartad, silandia, govia, pabil, tacuro, peratro, rememión, cártil, pimaón, rescano, crosa, cecetra, cacirro, reimaón, epepinio, sonfento, cancecto, canvial, infionso, canve, nocuto, salchesa, gerte, illecio, homple, pable, bumar, asamo, mumbo, ticeno, magle, trapimo, hinte, gotre, nomple, siampo, dercad.
